# The treatment of spleen injuries: a retrospective study

**DOI:** 10.1186/s13049-015-0163-6

**Published:** 2015-10-29

**Authors:** Trond Dehli, Anna Bågenholm, Nora Christine Trasti, Svein Arne Monsen, Kristian Bartnes

**Affiliations:** Department of Gastrointestinal Surgery, University Hospital of North Norway, Tromsø, 9038 Norway; Department of Radiology, University Hospital of North Norway, Tromsø, Norway; Department of Anesthesiology, Helgelandsykehuset, 8801 Sandnessjøen, Norway; Department of Cardiothoracic and Vascular Surgery, University Hospital of North Norway, Tromsø, Norway; Institute of Clinical Medicine, The Arctic University of Norway, Tromsø, Norway

**Keywords:** Spleen, Trauma, General surgery, Clinical coding, Interfacility transfer, Abbreviated injury scale

## Abstract

**Background:**

Hemorrhage after blunt trauma is a major contributor to death after trauma. In the abdomen, an injured spleen is the most frequent cause of major bleeding. Splenectomy is historically the treatment of choice. In 2007, non-operative management (NOM) with splenic artery embolization (SAE) was introduced in our institution. The indication for SAE is hemodynamically stable patients with extravasation of contrast, or grade 3–5 spleen injury according to the Abbreviated Organ Injury Scale 2005, Update 2008. We wanted to examine if the introduction of SAE increased the rate of salvaged spleens in our trauma center.

**Method:**

All patients discharged with the diagnosis of splenic injury in the period 01.01.2000 – 31.12.2013 from the University Hospital of North Norway Tromsø were included in the study. Patients admitted for rehabilitation purposes or with an iatrogenic injury were excluded.

**Results:**

A total of 109 patients were included in the study. In the period 2000-7, 20 of 52 patients were splenectomized. During 2007-13, there were 6 splenectomies and 24 SAE among 57 patients. The reduction in splenectomies is significant (*p* < 0.001). There is an increase in the rate of treated patients (splenectomy and SAE) from 38 to 53 % in the two time periods, but not significantly (*p* = 0.65).

**Conclusion:**

The rate of salvaged spleens has increased after the introduction of SAE in our center.

**Trial registration:**

The study is registered at www.clinicaltrials.gov with the identification number NCT01965548.

## Background

Injuries account for 12 % of the global disease burden, and are the leading cause of life-years lost in people under the age of 44 years [1]. Hemorrhage is the cause of 30–40 % of these deaths [1]. For bleeding patients reaching a hospital alive, damage control surgery is often performed, with emphasis on the control of bleeding and contamination, followed by definitive surgery after resuscitation in the intensive care unit [2].

In blunt abdominal trauma, the spleen is the most frequently injured organ [3]. Historically, splenectomy has been the treatment of choice. For exsanguinating patients, open splenectomy is still the proper choice of treatment if the spleen is a significant source of bleeding. However, for hemodynamically stable patients with splenic injury, non-operative management (NOM) is an alternative, assuming they have no other indication for laparotomy. In addition to avoiding a laparotomy, the greatest advantage of NOM is the preservation of splenic function, including full immune competence and avoiding the increased risk of septicaemia [4, 5]. NOM includes observation and/or splenic artery embolisation (SAE). In Norway, the first hospital to introduce SAE was Oslo University Hospital - Ullevål, Oslo in 2002 [6]. At University Hospital North Norway Tromsø (UNN) the first SAE was performed in 2007.

The indications for NOM with or without SAE varies between trauma centers [7]. The assessment of the indication for SAE in hemodynamic stable patients includes a grading of the spleen injury. The present grading system was published in 1994 and is based on the surgeons operative findings, independent of extravasation of contrast and hemoperitoneum seen on CT examinations [8, 9]. Today the grading is based mainly on computer tomography (CT) findings; the majority of patients are examined with CT before treatment decisions are made.

The Norwegian trauma system has two levels of hospitals, trauma centers and the acute care hospitals. SAE is a potential treatment option also for hemodynamically stable patients transferred from an acute care hospital to a trauma center. However, little is published about SAE in transferred trauma patients. A search in PubMed with the terms “splenic AND injury AND transfer” yielded only one paper describing six cases [10].

The aim of the study is to describe the treatment of splenic injuries and the potential changes in management after the introduction of SAE in the regional trauma center of the University Hospital of North Norway. We have also described if SAE is a treatment option for patients transferred from acute care hospitals to the trauma center, and hence, increase the possibility of saving the spleen in transferred patients.

The grading of splenic injuries is a major contributor to the indication for NOM. As the injury grading tool is originally based on surgical findings and grading today is mainly based on CT-findings, we have included an analysis of the inter-observer agreement of the spleen injury grading as part of the study.

## Materials and method

### Study location

UNN is a University Hospital with a primary catchment population of 100.000, and is the regional hospital and trauma center for a total of 480 000 people in North Norway. There are 9 acute care hospitals in North Norway admitting trauma patients, which transfer trauma patients to the trauma center after initial stabilization if necessary. In 2007 a treatment algorithm for spleen injuries was introduced. The algorithm included SAE in all hemodynamically stable patients with extravasation of contrast , and all patients with spleen injuries grade 3–5 independent of extravasation of contrast and amount of hemoperitoneum [8, 9].

### Design

The study is a retrospective, observational study.

### Identification of patients and inclusion-/exclusion criteria

All patients (including children) admitted or transferred to UNN in the period of 01.01.2000 – 31.12.2013 and with the discharge diagnosis S36.0 Splenic injury (ICD-10) [11] were included. Exclusion criteria were no injury (coding error), iatrogenic injury and admittance or transfer for rehabilitation purposes.

### Data collection

Data was collected from the electronic patient records at UNN, including radiological images and scanned or transferred documents from referring hospitals.

### Classification of injuries

All injuries were classified according to the Abbreviated Injury Scale (AIS) [8] and the extent of injuries are classified with the Injury Severity Scale (ISS) [12] by an authorized registrar.

The splenic injuries were classified retrospectively according to AIS and Organ Injury Scale (OIS) [8, 9]. The injuries of patients splenectomized without a preoperative CT-scan were also classified according to AIS and OIS after the surgeon’s description. If there were no CT-scan available for classification and no operative description, other available data on the injury was used for grading. All CT-scans were assessed retrospectively by two consultant radiologists independently. One radiologist is an approved AIS registrar and the other is an interventional radiologist. Any divergences were resolved by common assessment. The radiologists also classified the splenic vascular injuries (extravasation of contrast and pseudoaneurism) and the extent of bleeding in 0–5 compartments intraperitoneal from the spleen and other organ injuries (hematoma in 0–5 compartments) [13, 14].

### Parameters

The patients are described with age, sex, mechanism of injury, classification and extent of injuries with AIS and ISS and if there had been a transfer from other hospitals [8, 12].

The primary outcome parameter is the type of splenic trauma treatment performed at the acute care hospital and at UNN. The alternatives are non-operative treatment (NOM), non-operative treatment (NOM) with splenic artery embolisastion (SAE), laparotomy with splenectomy or any combination of the three alternatives.

Secondary outcome parameters include complications within < 30 days after SAE or splenectomy, 30 day mortality, length of hospital stay in total and in the intensive care unit, physiologic parameters and other emergency procedures. Physiologic parameters included systolic blood pressure (SBP) on admission, heart rate (HR) on admission, hemoglobin in g/dl (Hgb) on admission and if they were transfused with one unit of erythrocytes or more during the hospital stay. Emergency interventions includes damage control thoracotomy, damage control laparotomy, extraperitoneal packing of the pelvis, revascularisation of an extremity, craniotomy, insertion of intracranial pressure bolt, chest tube insertion and primary stabilization of fractures (external fixation).

### Statistical methods

Data are presented as frequency, mean, median with interquartile range and percentage. The frequency of splenectomies between patients included before and after 2007 is compared using Pearson’s chi-square test. The inter-observer grading scores for the spleen injury were analyzed with weighted kappa. A kappa value of 0.40–0.59 was considered a moderate agreement; a value of 0.60–0.79 was considered good and a value of 0.80–1.00 was an almost perfect agreement [15]. Significance is assumed for *p* < 0.05.

### Sample size calculation

We expected to include 10 patients per year in this study bases on a previous study [16]. That gives a total of approximately 130 patients, with half of the patients in the period 2000-6 and half of the patients in the period 2007-13. Approximately 25 % of the splenic trauma patients require a surgical intervention in order to stop the bleeding [17]. Before 2007 open surgery was the only option, in 2007 and later SAE became an option and is used in approximately half of patients in need of surgical intervention^14^. Based on the figures mentioned, we anticipated that the number of splenectomies per year should be reduced by 50 % after 2007. With a power of 80 % and a significance level of *p* < 0.05, the sample size required to detect such a difference is 124 patients in total [18, 19].

### Ethics

The study was approved by The Norwegian Data Protection Authority, approval from the Regional Medical Research Ethical Committee was not necessary. The study is registered at www.clinicaltrials.gov with the identification number NCT01965548.

## Results

**Fig. 1:**
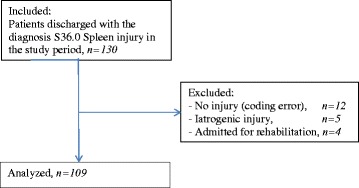
Patient flow

A total of 109 patients with splenic injury were included in the study, see Fig. 1. Of the 88 men and 21 women with a mean age of 32 years, 97 % had sustained a blunt trauma and 3 % a penetrating trauma. Injuries related to traffic accidents accounted for 55 % of cases, falls 31 %, 6 % were injured in accidents with snowmobiles, 5 % were hit by a blunt object, and 2 % were penetrating trauma. The median ISS for all patients was 16 (9, 21).

Physiologic parameters and ISS are given in Table 1 according to treatment of the spleen injury before and after the implementation of NOM with SAE in 2007. The mean hospital stay was 7.5 days, including 1.5 days in the intensive care unit. There were no differences in length of stay according to the given treatment.

**Table 1:** Physiologic data and Injury Severity Score (ISS) in 109 patients with splenic injury

	2000-6		2007-13		
Treatment of the spleen injury	NOM	Splenectomy	NOM	Splenectomy	NOM with SAE
Number of patients	32	20	27	6	24
Systolic blood pressure on admission, median with interquartile range	123 (107,130)	103 (70, 120)	124 (117, 136)	105 (76, 115)	120 (100, 130)
Heart rate on admission, median with interquartile range	85 (75,100)	100 (80, 110)	95 (80, 105)	110 (71, 142)	90 (75, 100)
Hemoglobin (g/dl) on admission, median with interquartile range	12.7 (11.2, 13.9)	11.3 (10.1, 12.4)	11.9 (10.8,3.2)	12.0 (11.5, 15.1)	11.7 (10.3, 13.6)
Transfusion^a^,number of patients	5	13	10	6	9
Injury Severity Score, median with interquartile range	13 (8, 20)	22 (16, 28)	16 (10, 18)	16 (10, 31)	16 (10, 18)

^a^ transfusion of 1 or more units of erythrocytes during the hospital stay

Five patients (4.6 %) died shortly after arrival. They had ISS ranging from 38 to 48. Two patients had head injuries as primary cause of death, and three patients died from massive hemorrhage from multiple injuries.

Of the 109 included patients, 96 (88%) were assessed as hemodynamically stable and had a CT scan (including one magnetic resonance imaging scan instead of CT) of the injured spleen. The inter-rater reliability grading of the spleen done by the two radiologists separately was moderate in agreement (kappa = 0.43, *p* < 0.001) before consensus was reached. 13 patients were either considered hemodynamically unstable or did not have a CT-scan of the splenic injury, and the grading is based on operative description (10 patients), ultrasound examination (1 patient), autopsy (1 patient) and primary radiological description of the splenic injury with no access to the CT-scan taken at the time (1 patient).

Details about the treatment given including AIS code of the spleen injuries are given in Table 2. Among the 24 patients who had angiography performed, 23 were embolised. Of the 24, 14 had contrast extravasion on angiography. No patient was later splenectomized due to failure to control the bleeding, but one patient was re-embolised due to continuous bleeding. There were no other complications after SAE.

**Table 2:** An overview of the treatment given to 109 patients with splenic injury according to AIS-grading of the splenic injury

		2000–2006		2007–2013		
	Number of patients	NOM	Splenectomy	NOM	Splenectomy	NOM with SAE
AIS 2–5	109	32	20	27	6*	24
AIS 2	37	18	1	15	1	2^a^
AIS 3	45	12	8	9	4	12
AIS 4	26	2	10	3	1	10
AIS 5	1	0	1	0	0	0

*AIS* abbreviated injury scale, *NOM* non-operative management, *SAE* splenic artery embolization

*significant reduction in splenectomies after 2006 (*p* < 0.001)

^a^ one patient had angiography without embolization

In the 96 patients with a CT-scan of the injury the spleen injuries were also assessed according to vascular injury and extent of intraperitoneal bleeding. An overview of these characteristics and corresponding treatment is given in Tables 3 and 4.

**Table 3:** An overview of the treatment given to 91 patients with a CT scan with contrast of a splenic injury according to the presence of vascular injury (contrast extravasation or pseudoaneurism)

		2000-6		2007-13		
Treatment of the spleen injury	Number of patients	NOM	Splenectomy	NOM	Splenectomy	NOM with SAE
No contrast extravasation on CT	71	20	7	25	2	17
Contrast extravasation on CT	17	3	8	0	5	1
Pseudoaneurism	3	1	0	1	0	1

*NOM* non-operative management, *SAE* splenic artery embolization

**Table 4:** An overview of the treatment given to 95 patients with a complete CT of the abdomen and pelvis with contrast after splenic injury according to the presence of hematoma in 0 to 5 compartments in the abdomen and pelvis

		2000-6		2007-13		
	Number of patients	NOM	Splenectomy	NOM	Splenectomy	NOM with SAE
No hematoma	19	10	1	7	0	1
1 compartment	17	6	0	11	0	0
2 compartments	9	2	0	2	1	4
3 compartments	21	5	2	4	1	9
4 compartments	1	0	0	0	0	1
5 compartments	28	3	12	2	2	9

*NOM* non-operative management, *SAE* splenic artery embolization

There were 26 children aged 16 years or younger. The median ISS was 16 (9, 20), of these 12 were treated with NOM, 10 were treated with NOM and SAE, and four were splenectomized. None of the children died.

A total of 35 patients were transferred from other hospitals. After 2007, nine of these were transferred specifically for SAE. For all transferred patients, the median time from injury to arrival at the trauma center was 11 h (IQR 7, 24). Three patients had splenectomy performed before transfer. Among the remaining 32 patients, 18 were treated with NOM, 11 had NOM with SAE and three had splenectomy after transfer.

The main finding is a significant reduction in splenectomies after the introduction of SAE in 2007 (*p* < 0.001) (Table 2). The proportion of patients with spleen injuries who were treated invasively (splenectomy or SAE), increased from 38 % in the period 2000-6 to 51 % during 2007-13 but this finding was not significant (*p* = 0.65).

Other emergency interventions included laparotomy (including splenectomy, *n* = 28), chest tube insertion (*n* = 24), insertion of intracranial pressure bolt (*n* = 5), thoracotomy (*n* = 4), and external fixation of fractures (*n* = 2).

## Discussion

Over a 14-year period, approximately eight patients per year were admitted with a spleen injury in our hospital. After the introduction of SAE in 2007, the proportion of open surgery with splenectomy has decreased. In addition, SAE has been used successfully in transferred patients. The overall complication rate is low. The study shows that after the introduction of SAE, the rate of salvaged spleens increased in our trauma center, and that SAE is an option for patients primarily admitted in hospitals without an angiographic treatment option. Within a regional trauma system with capabilities for transfer, this might reduce the number of splenectomies in hospitals without an angio-intervention capability.

A limitation of the study is that our hospital admits few patients with spleen injuries, making it possible for small inconsistencies in grading and management to influence the data. The grading of spleen injuries in this study is done by two radiologists to improve the precision of the classification. The long study period also allows for other changes in the management of spleen injuries to occur and possibly influencing our results, such as increased awareness of NOM, the introduction of massive transfusion protocols, early treatment with tranexamic acid in trauma and the development of a regional trauma system [20, 21].

The grading of spleen injuries follows OIS revised 1994, and the AIS 2005 upgrade 2008 is based on OIS [8, 9]. The OIS grading scale of the splenic injury is a short description in text based on operative findings, with no pictures to illustrate the grading and with no change in the text since 1994. The most used grading system for splenic injuries is not adjusted to the fact that almost all grading is done after translating the CT pictures to the written grading scale. The grading itself might therefore be difficult, with the potential for inconsistencies [22]. This might influence the choice of treatment in spleen injuries, and also the results presented in studies on spleen injuries. A more extensive grading tool including CT findings would ensure a more consistent grading between different radiologist and centers. In addition, the OIS do not take into account extravasation of contrast, vascular injuries or hematoma around the spleen and in the abdomen. A grade 2 spleen injury with contrast extravasation might be a more severe injury than a grade 3 spleen injury without hematoma or signs of vascular injury. Extravasation of contrast is an indication for SAE independent of injury grade. Two new grading systems of spleen injuries incorporating vascular injuries have been proposed, but has not achieved widespread use [13, 14]. As this study suggests, the presence of vascular injuries and the extent of intraperitoneal hematoma does seem to influence the choice of treatment (Tables 2 and 3).

The indication for SAE in patients with spleen injuries varies between centers. The variation in treatment is both in total proportion of patients treated and in which spleen injury grade different treatment options are used [7]. Injury grade 3 or higher has been identified as a risk factor for failure of conservative treatment without SAE [4], but a study from Norway concludes that SAE in grade 3 splenic injuries does not seem justified [6]. In this study, a grade 3 spleen injury is treated with SAE, partly with support in the literature and partly to compensate for a limited experience with the procedure and also the grading of spleen injuries. The results are good with fewer complications than reported in other studies [23], but they also indicate the need for an evaluation of the indication for SAE of spleen injuries in our center. In addition, the use of SAE requires radiation. Long-term consequences for such radiation are under debate, there might be a harmful effect if SAE is done when it is not medically justified [24]. This should be taken into consideration in the treatment algorithms.

## Conclusion

In conclusion, the study shows that after the introduction of NOM with SAE, the salvage rate of injured spleens has increased in a center with a low volume of such injuries. In addition, SAE is also an option for patients primarily admitted in hospitals without an angio-intervention capability, assuming that the patients are physiologically stable and transfer is available. There is a need for an update on the grading system of splenic injuries, which include CT-findings of the spleen, splenic vessels and amount of intraperitoneal hematoma.
